# Association of Gestational Age at Birth With Risk of Perinatal Mortality and Special Educational Need Among Twins

**DOI:** 10.1001/jamapediatrics.2019.6317

**Published:** 2020-03-09

**Authors:** Sarah Murray, Daniel MacKay, Sarah Stock, Jill Pell, Jane Norman

**Affiliations:** 1 MRC Centre for Reproductive Health, Queen’s Medical Research Institute, University of Edinburgh, Edinburgh, Scotland; 2 Institute of Health and Wellbeing, University of Glasgow, Glasgow, Scotland; 3 Faculty of Health Sciences, University of Bristol, Bristol, England

## Abstract

**Importance:**

Twin pregnancies account for 3% of live births but experience substantially more perinatal morbidity and mortality than singleton pregnancies. Optimizing the timing of birth is a key strategy in improving twin pregnancy outcomes. Current UK and US policies are based on observational studies of perinatal mortality and not on longer-term effects. The association of timing of birth with long-term childhood outcomes among twins is uncertain.

**Objective:**

To identify the optimal gestation week for birth of twin infants by calculating the week of birth associated with the lowest risk of short-term and long-term adverse outcomes (perinatal mortality and special educational need [SEN] at school).

**Design, Setting, and Participants:**

This population-based, data-linkage cohort study included 43 133 twin infants born at a gestational age of 34 weeks onward between January 1, 1980, and December 31, 2015, in Scotland. The data were analyzed from June 1, 2017, to March 1, 2019.

**Exposures:**

Gestational age (in weeks) at the time of birth.

**Main Outcomes and Measures:**

The primary outcomes were extended perinatal mortality and a record of SEN (≥1 of intellectual disabilities, dyslexia, physical or motor impairment, language or speech disorder, autistic spectrum disorder, and social, emotional, or behavioral difficulties) at school (children aged 4-18 years). To infer the consequence of the gestational age at birth, clinical outcomes of twin infants born at each week of gestation from 34 weeks onward were compared with those of twin infants remaining in utero thereafter.

**Results:**

Of the total 43 133 twin infants included in the study, 21 696 (50.3%) were females. Although maternity records were available for all infants, 9519 sex-discordant twin children were linked to their educational data, of whom 1069 (13.8%) had a record of SEN. Compared with twins remaining in utero (n = 26 172), birth at any gestational age from 34 to 37 weeks was associated with increased odds of perinatal death (ie, adjusted odds ratio [AOR], 1.99; 95% CI, 1.53-2.69 at 36 weeks [n = 8056]) and increased risk of SEN at school (AOR, 1.39; 95% CI, 1.11-1.74, for birth at 36 weeks compared with 37 weeks). In a competing risk analysis, the risks of stillbirth and neonatal death were balanced at 37 weeks (risk difference, 2.05; 95% CI, 0.8-3.3).

**Conclusions and Relevance:**

The findings of this study suggest that, in the absence of a medical complication, twins should not be routinely delivered before 37 completed weeks of gestation. These findings may help optimize shared decision-making around the timing of twin birth.

## Key Points

**Question:**

What is the association of gestational age at the time of birth of twins with the risk of perinatal mortality and special educational need and what is the optimal week for the birth?

**Findings:**

This population cohort study of 43 133 twins using record linkage of their maternity and educational data found that delivery before 37 weeks of gestation was associated with an increased risk of perinatal death and special educational need at school.

**Meaning:**

The findings of this study suggest that twin infants should not be delivered before 37 completed weeks of gestation in the absence of medical complications.

## Teaser

This cohort study examines the association of gestational age at the time of birth of twins with the risk of perinatal mortality and special educational need in school by analyzing data on 43 133 twins born in Scotland between 1980 and 2015.

## Introduction

The rate of birth of twins has been increasing, in part owing to assisted reproduction methods, with 24% of these procedures resulting in twins.^1,2^ Twin pregnancies account for only 3% of live births but account for 15% of neonatal and special care baby unit admissions.^3^ These admissions and the increased cesarean delivery rate together result in a greater health care expenditure of twins, estimated to be 3 times that of singletons.^4^ Twin pregnancies are also associated with higher perinatal mortality, owing to higher rates of stillbirth (infants born without signs of life after 24 weeks of gestation) and neonatal death (infants born alive but who die within 28 days).^2^ Thus, optimizing the timing of birth is a key strategy in reducing perinatal mortality and is a research priority.^2,5^ The optimum timing of birth of twins remains uncertain. Randomized controlled trials on the timing of birth have not been adequately powered to draw definitive conclusions^6,7^; therefore, observational studies have been used to inform current policies for twins (National Institute for Health and Clinical Excellence in the UK [NICE]^2^ and the Society for Maternal and Fetal Medicine [SMFM] in the US^8^). However, these observational studies have some methodological weaknesses; many used live births as the denominator rather than population at risk (which includes ongoing pregnancies), and they do not adjust for the clustering of twins.^9,10^ A systematic review of observational and randomized studies concluded that, to minimize perinatal death, birth should be considered from 37 weeks in dichorionic pregnancies and 36 weeks in monochorionic pregnancies.

Data from singletons illustrate the need to consider the association of timing of birth with long-term neurodevelopment outcomes (with performance at school as a surrogate). In uncomplicated singleton pregnancies, perinatal mortality is lowest at 38 to 39 weeks,^11,12,13^ but the odds of having a record of special educational need (SEN) at school (intellectual, physical, or motor impairment) is lowest at 41 weeks of gestation.^14^ Hence, although perinatal death could be reduced by earlier delivery, this may increase neurodevelopmental compromise.^15^

The present study’s objective was to explore the association between gestational age at birth of twins and short- and long-term childhood outcomes.

## Methods

### Study Population

This population-based cohort study assessed twin pregnancies delivered at a gestational age of 34 weeks or more between January 1, 1980, and December 31, 2015. The study was approved by the NHS (National Health Service) Scotland Public Benefit and Privacy Panel for Health and Social Care, and the South East Scotland Research Ethics Committee. All data were nonidentifiable, and the Public Benefit and Privacy Panel for Health and Social Care waived the need for patient consent. A data processing and sharing agreement was produced between the University of Edinburgh, Edinburgh, Scotland, and the NHS Information Services Division.

### Databases

Data were obtained from the Scottish Morbidity Record 02 (SMR02), the Scottish Stillbirth and Infant Death Survey (SSBID), and the Scottish Exchange of Educational Data (ScotXed). The study population was derived from the SMR02 maternity database, which collects data on maternal, obstetric, and neonatal outcomes. The SSBID database contains information on stillbirths and infant deaths that are registered, with registration mandated by law. The ScotXed contains details on the pupil census and has been conducted annually since 2005 by all local authority–run schools. The information includes whether a child has an SEN. The SMR02 database is subjected to regular quality assurance checks and has been more than 99% complete since 1980.^16^ With the use of SMR02 data as the base population, the educational data from the ScotXed database was record-linked to the SMR02 by the Information Services Division. The linkage method has been described previously.^17^ The follow-up study of the educational outcomes was limited to sex-discordant twins because we could not ascertain whether the correct twin records were linked for same-sex twins.

### Inclusion and Exclusion Criteria

Women were included if they had a twin birth at or after 34 weeks of gestation. Births were excluded if there was a congenital anomaly, gestational age at birth was recorded as missing or as longer than 44 weeks, maternal age was less than 10 years, parity was missing or more than 14, birth weight was greater than 5000 g, or fetal sex was recorded as missing. For the follow-up study, we excluded individuals whose age was recorded as younger than 4 years or older than 19 years.

### Outcomes, Exposures, and Covariates

The exposure of interest was gestational age at birth. In SMR02, the units for gestational age at birth are completed weeks of gestation, calculated using the estimated date of delivery. This variable has been described previously and is considered accurate and of high quality,^14^ with more than 95% of women having had gestational age confirmed by ultrasonography since the early 1990s.^18^ We compared birth at a particular week of gestation with ongoing pregnancies (ie, women who go on to deliver at a later gestation by any mode of onset; this method has been described previously^11,13^).

The 2 primary outcomes were extended perinatal mortality and a record of SEN at school. Extended perinatal mortality was defined as combined stillbirth or neonatal death. SEN is defined by the UK Department of Education as being unable to benefit fully from school without help beyond that normally given to schoolchildren of the same age.^19^ In the present study, SEN was defined as a record of any of the following: intellectual disabilities, dyslexia, physical or motor impairment, language or speech disorder, autistic spectrum disorder, and social, emotional, or behavioral difficulties.

The secondary outcomes studied included neonatal morbidity (a composite measure of morbidity defined as an infant with a low Apgar score [<7 at 5 minutes after birth], who required admission to a neonatal, special care baby, or neonatal intensive care unit, or who required assisted ventilation). An exploratory analysis of academic achievement at school and leaver destination was performed. Highest academic attainment was derived from the number of examinations attained at each level of the Scottish Credit and Qualifications Framework^20^ and converted into the following: poor educational attainment (low [≥1 at level 2, <5 at level 3, or <2 at level 4] and basic attainment [≥5 at level 3, ≥2 at level 4, or ≤4 at level 5]), high educational attainment (broad attainment [>7 at level 4, >5 at level 5, or <3 at level 6], and highest attainment [>1 at level 7 or ≥3 at level 6]). School leaver destination was defined as a dichotomous variable of higher or further education, employment, or training, or unemployment.

The following variables were considered potential confounders and were included in the multivariable regression analyses: maternal age at birth of twins (≤20, 21-30, 31-40, and >40 years), parity during the index pregnancy (para 0 or para ≥1), year of birth (1981-1985, 1986-1990, 1991-1995, 1996-2000, 2001-2005, 2006-2010, or 2011-2015), area socioeconomic deprivation quintile of postcode of residence (defined by Carstairs 2001 quintiles: 1 [most affluent] to 5 [most deprived]^21^), gestation-specific and sex-specific birth weight centiles (<3, 4-10, 11-90, 91-97, or >97), fetal sex (male or female), smoking status at booking (current smoker or nonsmoker), and maternal height (<150, 150-154, 155-159, 160-164, 165-169, 170-174, or >175 cm).

### Statistical Analyses

The data were analyzed from June 1, 2017, to March 1, 2019. Summary statistics were derived and compared by the outcome of perinatal death. To assess the risk of perinatal death at each week of gestation compared with ongoing pregnancies, univariate and multivariate generalized estimating equation (GEE) analyses were performed. The user-written quasi-likelihood under the independence model criterion (QIC) statistic was used to compare different correlation structures.^22^ The structure with the lowest trace QIC was selected. To assess for the consequence of medically indicated deliveries, an interaction term was included in the GEE model and compared with the model without interaction. The indication for delivery is not recorded in SMR02, unlike medical conditions in pregnancy (recorded using the *International Classification of Diseases, Ninth Revision* [*ICD-9*] and *International Statistical Classification of Diseases and Related Health Problems, Tenth Revision* [*ICD-10*]).^23^ Medically indicated delivery was defined as at least 1 of the following conditions: hypertensive disease (*ICD-10* code O10), diabetes mellitus (*ICD-10* code O24), small for gestational age (*ICD-10* code P05), thromboembolic disease (*ICD-10* code O22), liver disorders (*ICD-10* code O26), antenatal investigation of abnormality (*ICD-10* code O42), and poor obstetric history (previous stillbirth or neonatal death *ICD-10* code O09). A competing risk analysis was performed to assess the risk of delivery vs expectant management at a particular week. The competing risk of perinatal death at a given gestational week was defined as the difference between the risk of stillbirth and risk of neonatal death for deliveries in that week, thus providing a direct measure of benefit or harm. Univariate and multivariate GEE analyses were performed to assess the associations between gestational age at birth and a record of SEN at school. Leaver status and academic achievement were analyzed using multivariable logistic regression modeling.

Missing values for maternal height, smoking status, and deprivation category were created using multiple imputation by chained equations using ICE in Stata.^24^ All covariates and outcomes were included in the imputation, and 30 imputed data sets were created. The multivariable models were fitted to each imputed data set, and a pooled result was obtained. A sensitivity analysis of complete cases against the imputed data sets was performed.

Chorionicity is not recorded in SMR02, but because chorionicity is associated with the risk of perinatal mortality (2-fold higher perinatal mortality in monochorionic vs dichorionic twins^25^), a subgroup analysis of only dichorionic twins (identified by sex discordance) was performed.

Population-attributable fractions^26^ were estimated using individuals with complete data to assess the proportion of perinatal death that was potentially explained by gestation at birth.

### Sensitivity Analyses

To assess the consequences of pregnancies complicated by 1 perinatal death that occurred more than 1 week before birth (ie, a stillbirth in a different week than the delivery week), a sensitivity analysis was performed excluding pregnancies with extreme birth weight discordance at birth. Specifically, if there was 1 fetal death and extreme birth weight discordance (defined as a difference in birth weight of >40% [calculated using the difference in the mean twin birth weight in the Scottish population at 28 and 32 weeks in the cohort]), we assumed that the intrauterine fetal death likely occurred more than 1 week before birth and that inclusion of this twin pair would lead to an overestimation of stillbirth.

A sensitivity analysis was performed excluding medically indicated deliveries for both perinatal death and SEN. *P* values for hypothesis tests were 2-sided and set at *P* < .05. All analyses were undertaken using Stata, version 14.1 (StataCorp).

## Results

**Figure 1.**
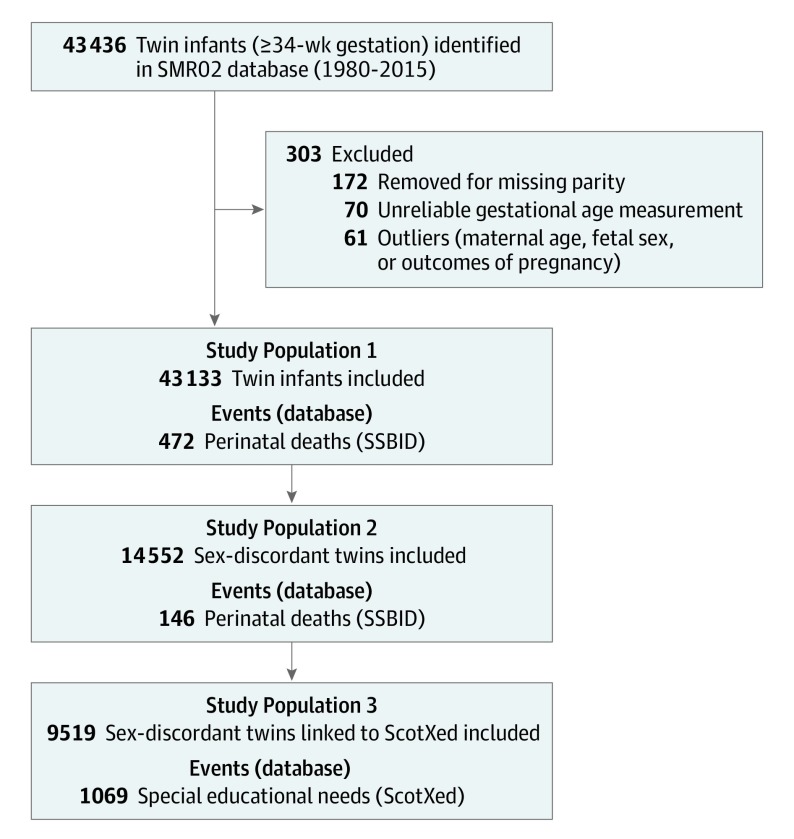
Derivation of the Study Cohort (Derivation of the Study Populations for the Different Outcomes) SMR02 indicates Scottish Morbidity Record 02; SSBID, Scottish Stillbirth and Infant Death Survey; and ScotXed, Scottish Exchange of Educational Data.

The SMR02 database contained 43 436 twin infants born in Scotland between January 1, 1980, and December 31, 2015, with 43 133 infants eligible for inclusion in the analysis. Of them, 21 696 (50.3%) were female infants. A total of 472 perinatal deaths, 354 stillbirths, and 118 neonatal deaths were recorded. The process of deriving the study cohort is outlined in Figure 1.

The largest proportion of twins were delivered between 37 and 38 weeks’ gestation (21 057 [48.8%]), and 16 961 twin infants (39.3%) were born preterm. In total, 26 578 mothers (61.1%) were between ages 25 and 35 years at the time of delivery, and 24 576 (57.0%) were primigravid. Table 1 gives the cohort characteristics. Data were missing for deprivation category (782 [1.8%]), maternal height (6289 [14.6%]), and maternal smoking (14 805 [34.3%]).

**Table 1.** Baseline Demographics of the Population of 43 133 Twins Born in Scotland and the Odds of Perinatal Death

Variable	No. of Twin Births (%)	Perinatal Deaths, No. (%)	Crude OR (95% CI)	*P* Value
Gestation, wk				
34	3774 (8.8)	85 (2.2)	3.25 (2.38-4.43)	<.001
35	5131 (11.9)	85 (1.7)	2.37 (1.74-2.34)	
36	8056 (18.7)	103 (1.3)	1.82 (1.36-2.45)	
37	10 925 (25.3)	77 (0.7)	1 [Reference]	
38	10 132 (23.5)	73 (0.7)	1.02 (0.74-1.41)	
39	3261 (7.6)	29 (0.9)	1.26 (0.82-1.94)	
≥40	1854 (4.3)	20 (1.1)	1.54 (0.94-2.52)	
Missing	0			
Sex				
Male	21 437 (49.7)	247 (1.2)	1.1 (0.93-1.33)	.25
Female	21 696 (50.3)	225 (1.0)	1 [Reference]	
Missing	0			
Parity				
Primiparous	18 287 (42.2)	202 (1.1)	1.02 (0.85-1.22)	.86
Nulliparous	24 576 (57.6)	270 (1.2)	1 [Reference]	
Missing	0			
Maternal age, y				
<20	2235 (5.2)	29 (1.3)	1.27 (0.85-1.90)	.77
20-25	7610 (17.6)	91 (1.2)	1.17 (0.89-1.52)	
26-30	13 336 (30.9)	137 (1.0)	1 [Reference]	
31-35	13 242 (20.7)	144 (1.1)	1.06 (0.84-1.43)	
36-40	5915 (13.7)	61 (1.0)	1.01 (0.74-1.36)	
>40	795 (1.8)	10 (1.3)	1.23 (0.85-1.90)	
Missing	0			
Year of birth				
1980-1985	6140 (14.2)	131 (2.1)	4.31 (2.89-6.42)	<.001
1986-1990	5692 (13.2)	73 (1.3)	2.57 (1.68-3.93)	
1991-1995	6277 (14.6)	73 (1.2)	2.33 (1.52-3.56)	
1996-2000	6194 (14.4)	65 (1.0)	2.10 (1.36-3.24)	
2001-2005	5900 (13.7)	42 (0.7)	1.42 (0.89-2.27)	
2006-2010	6970 (16.2)	58 (0.8)	1.66 (1.07-2.58)	
2011-2015	5960 (13.8)	30 (0.5)	1 [Reference]	
Missing	0			
Deprivation quintile^a^				
1	8568 (19.9)	83 (1.0)	1 [Reference]	.15
2	7908 (18.3)	94 (1.2)	1.23 (0.91-1.65)	
3	8199 (19.0)	79 (1.0)	1.00 (0.73-1.36)	
4	8332 (19.3)	85 (1.0)	1.05 (0.78-1.43)	
5	9344 (21.7)	120 (1.3)	1.33 (1.03-1.76)	
Missing	782 (1.8)			
Birth weight, centile				
1-3	1337 (3.1)	125 (9.4)	22.9 (17.7-29.7)	<.001
4-10	3078 (7.1)	66 (2.1)	4.9 (3.6-6.6)	
11-20	4360 (10.1)	37 (0.8)	1.9 (1.3-2.8)	
21-80	25 900 (60.0)	116 (0.4)	1 [Reference]	
81-90	4256 (9.9)	20 (0.5)	1.0 (0.7-1.69)	
91-97	2941 (6.8)	14 (0.5)	1.1 (0.6-1.9)	
98-100	1261 (2.9)	94 (7.4)	17.9 (13.6-23.7)	
Missing	0			
Smoking status at booking				
Smoker	5912 (13.7)	68 (1.2)	1.54 (1.16-2.05)	.003
Nonsmoker	22 416 (52.0)	168 (0.8)	1 [Reference]	
Missing	14 805 (34.3)			
Maternal height, cm				
<150	456 (1.2)	9 (2.0)	1.87 (0.94-3.71)	.03
150-154	2744 (7.4)	38 (1.4)	1.30 (0.90-1.88)	
155-159	6609 (17.9)	84 (1.3)	1.20 (0.90-1.58)	
160-164	10 982 (29.8)	117 (1.1)	1 [Reference]	
165-169	9265 (25.2)	83 (0.9)	0.84 (0.63-1.11)	
170-174	4923 (13.4)	49 (1.0)	0.93 (0.67-1.31)	
>174	1865 (4.3)	13 (0.7)	0.65 (0.37-1.16)	
Missing	6289 (14.6)			

Abbreviation: OR, odds ratio.

^a^ Area socioeconomic deprivation quintile of postcode of residence as defined using Carstairs 2001 quintiles, where 1 indicates most affluent and 5, most deprived.^21^

### Short-term Perinatal Outcomes According to Gestational Age at Birth

#### Primary Outcome

Outcomes of perinatal mortality by week of gestation compared with ongoing pregnancies are reported in Table 2. Compared with the twins remaining in utero (n = 26 172), birth of twins at any complete week from 34 to 37 weeks of gestation was associated with an increased risk of perinatal mortality (adjusted odds ratio [AOR], 2.59; 95% CI, 1.99-3.39 at 34 weeks [n = 3774]; AOR, 2.12; 95% CI, 1.63-2.76 at 35 weeks [n = 5131]; and AOR, 1.99; 95% CI, 1.53-2.69 at 36 weeks [n = 8056]). Birth at 37, 38, or 39 weeks of gestation was associated with no increased risk of perinatal mortality compared with that for the infants remaining in utero (AOR, 1.10; 95% CI, 0.81-1.51 at 37 weeks; AOR, 0.92; 95% CI, 0.61-1.38 at 38 weeks; and AOR, 0.77; 95% CI, 0.41-1.45 at 39 weeks). Gestation at birth before 37 weeks had a population-attributable fraction (PAF) of perinatal mortality of 34.2% (Table 2).

**Table 2.** Perinatal Mortality, Perinatal Morbidity, and PAF for Perinatal Mortality at Each Week of Gestation Compared With Remaining In Utero^a^

Week of Gestation	No. With Outcome/Total No. in Group (%)		OR (95% CI)				PAF
	Ongoing Pregnancies	Delivered	Unadjusted	*P* Value	Adjusted^b^	*P* Value	
**Perinatal Mortality**							
34	387/39 359 (1.0)	85/3774 (2.2)	2.32 (1.80 to 3.00)	<.001	2.59 (1.99 to 3.39)	<.001	0.101 (0.630 to 0.138)
35	302/34 228 (0.9)	85/5131 (1.7)	1.89 (1.45 to 2.44)	<.001	2.12 (1.63 to 2.76)	<.001	0.103 (0.543 to 0.149)
36	199/26 172 (0.8)	103/8056 (1.3)	1.69 (1.31 to 2.18)	<.001	1.99 (1.53 to 2.59)	<.001	0.138 (0.066 to 0.205)
37	122/15 247 (0.8)	77/10925 (0.7)	0.88 (0.65 to 1.18)	.40	1.10 (0.81 to 1.51)	.54	−0.0523 (−0.175 to 0.057)
38	49/5115 (1.0)	73/10132 (0.7)	0.75 (0.52 to 1.09)	.13	0.92 (0.61 to 1.38)	.52	−0.197 (−0.485 to 0.035)
39	20/1854 (1.1)	29/3261 (0.9)	0.82 (0.45 to 1.49)	.52	0.77 (0.41 to 1.45)	.41	−0.126 (−0.575 to 0.195)
**Perinatal Morbidity**							
34	11 712/39 359 (29.8)	3244/3774 (86.0)	14.45 (12.76 to 16.36)	<.001	16.23 (14.23 to 18.45)	<.001	0.142 (0.141 to 0.142)
35	8525/34 228 (24.9)	3187/5131 (62.1)	4.94 (4.56 to 5.36)	<.001	5.67 (5.21 to 6.17)	<.001	0.163 (0.162 to 0.164)
36	5431/26 172 (20.8)	3094/8056 (38.4)	2.38 (2.22 to 2.55)	<.001	2.77 (2.58 to 2.99)	<.001	0.167 (0.165 to 0.169)
37	2948/15 247 (19.3)	2483/10925 (22.7)	1.23 (1.14 to 1.33)	<.001	1.50 (1.38 to 1.63)	<.001	0.068 (0.065 to 0.071)
38	1087/6976 (15.6)	1861/8271 (22.5)	0.83 (0.75 to 0.93)	.001	1.01 (0.90 to 1.14)	.82	−0.099 (−0.107 to −0.908)
39	398/1404 (28.3)	689/2572 (26.8)	0.98 (0.82 to 1.17)	.82	1.05 (0.87 to 1.27)	.60	−0.010 (−0.023 to 0.002)

Abbreviations: OR, odds ratio; PAF, population-attributable fraction.

^a^ Perinatal morbidity was defined as a composite of an Apgar score less than 7, assisted ventilation, or admission to the neonatal unit.

^b^ Adjusted for maternal age and height, parity, sex of the infant, birth order, year of delivery, deprivation category, birth weight centile, and smoking.

**Figure 2.**
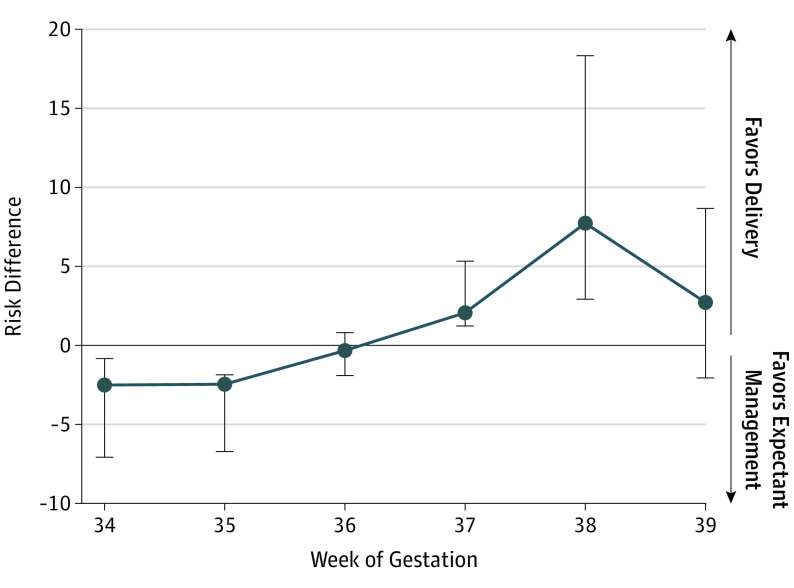
Competing Risk Analysis Results Data markers depict the risk difference per 1000 pregnancies; error bars, 95% CI.

In the competing risk analysis, the risk of stillbirth was significantly lower than the risk of neonatal death at 34 and 35 weeks of gestation (risk difference, −2.49 at 34 weeks and −2.43 at 35 weeks; Figure 2) but balanced at 37 weeks (risk difference, 2.05; 95% CI, 0.8-3.3). After 37 weeks, the risk of stillbirth significantly outweighed the risk of neonatal death (risk difference, 7.80; 95% CI, 4.86-10.55; Figure 2).

#### Secondary Outcomes

Table 2 provides the neonatal morbidity for twin infants delivered and those remaining in utero for each week of gestation. Twin infants born before 38 weeks had an increased risk of neonatal morbidity compared with that for infants remaining in utero (AOR, 16.23; 95% CI, 14.23-18.45 at 34 weeks; AOR, 5.67; 95% CI, 5.21-6.17 at 35 weeks; AOR, 2.77; 95% CI, 2.58-2.99 at 36 weeks; and AOR, 1.50; 95% CI, 1.38-1.63 at 37 weeks). Birth at 38 and 39 weeks’ gestation had no increased or decreased risk of neonatal morbidity compared with that for the infants remaining in utero.

### Sex-Discordant Twins

Results of the subgroup analysis of sex-discordant dichorionic twins (n = 14 755) were similar to the results for all twins: birth before 37 weeks was associated with an increased risk of perinatal death compared with that for the infants remaining in utero (AOR, 1.81; 95% CI, 1.28-2.55, eTable 1 in the Supplement).

### Long-term Educational Outcomes According to Gestational Age at Birth

#### Primary Outcome

A total of 9519 sex-discordant twin children were linked to their educational data, and 1069 (13.8%) had a record of SEN. An inverse linear association was observed between gestational age at birth and SEN (eFigure in the Supplement). Compared with birth at 37 weeks, children born at each week of gestation before 37 weeks had an increased risk of SEN at school (AOR, 1.35; 95% CI, 1.01-1.82 at 34 weeks; AOR, 1.35; 95% CI, 1.05-1.74 at 35 weeks; and AOR, 1.39; 95% CI, 1.11-1.74 at 36 weeks, Table 3). The risk of SEN did not change with birth between 38 and 39 weeks or after 40 weeks (Table 3). The overall rate of SEN and the rate at each gestational week was higher among twins than that previously reported among singletons^14^ (eTable 2 in the Supplement; only nonmedically indicated twin births were included in this table to compare with nonmedically indicated singleton births reported by MacKay et al^14^).

**Table 3.** Association Between Gestational Age at Birth and Special Educational Need, Leaver Status, and Academic Attainment

Gestational Age at Birth, wk	No. of Outcomes/ Total No. (%)	OR (95% CI)	
		Unadjusted	Adjusted^a^
Special educational need			
34	98/682 (14.4)	1.30 (0.97-1.74)	1.35 (1.01-1.82)
35	148/1005 (14.7)	1.34 (1.05-1.71)	1.35 (1.05-1.74)
36	235/1560 (15.1)	1.37 (1.10-1.71)	1.39 (1.11-1.74)
37	263/2301 (11.4)	1 [Reference]	1 [Reference]
38	239/2184 (10.9)	0.95 (0.77-1.18)	1.00 (0.80-1.24)
39	64/538 (11.9)	1.05 (0.75-1.47)	1.02 (0.73-1.44)
≥40	22/180 (12.2)	1.08 (0.64-1.82)	1.16 (0.69-1.96)
Leaver status (not employed at 6 mo)			
34	19/219 (8.7)	0.74 (0.41-1.34)	0.77 (0.42-1.43)
35	22/259 (7.8)	0.66 (0.38-1.15)	0.67 (0.38-1.19)
36	43/385 (10.0)	0.87 (0.56-1.36)	0.91 (0.59-1.43)
37	71/553 (11.4)	1 [Reference]	1 [Reference]
38	51/579 (8.1)	0.69 (0.45-1.04)	0.77 (0.51-1.18)
39	34/185 (15.5)	1.43 (0.87-2.36)	1.75 (1.06-2.87)
≥40	16/108 (12.9)	1.15 (0.60-2.21)	1.26 (0.65-2.49)
Poorer academic attainment			
34	19/223 (8.5)	1.14 (0.60-2.15)	1.07 (0.57-2.02)
35	28/290 (9.7)	0.99 (0.59-1.68)	0.95 (0.54-1.65)
36	30/426 (6.6)	1.51 (0.88-2.58)	1.48 (0.85-2.58)
37	62/646 (9.6)	1 [Reference]	1 [Reference]
38	48/616 (7.8)	1.26 (0.79-2.00)	1.17 (0.73-1.90)
39	20/219 (9.1)	1.06 (0.59-1.90)	0.91 (0.50-1.64)
≥40	7/80 (8.8)	1.11 (0.44-2.77)	1.07 (0.43-2.70

Abbreviation: OR, odds ratio.

^a^ Adjusted for maternal age and height, parity, sex of the infant, birth order, year of delivery, social class, birth weight centile, and smoking.

#### Secondary Outcomes

The exploratory analysis of academic attainment included 2551 twins. Gestational age at birth was not associated with low attainment at school (AOR, 1.07; 95% CI, 0.57-2.02 at 34 weeks compared with birth at 37 weeks; Table 3). The analysis of leaver destination included 2531 twins. Birth before 37 weeks was not associated with unemployment (AOR, 0.77; 95% CI, 0.42-1.43 at 34 weeks compared with birth at 37 weeks). Birth at 39 weeks was associated with an increased risk of unemployment compared with that for birth at 37 weeks (AOR, 1.75; 95% CI, 1.06-2.87).

### Sensitivity Analyses

The results were similar when the logistic regression models were run without imputation of missing values (eTable 3 in the Supplement). There was no evidence of interaction of medically indicated deliveries compared with all twin deliveries (likelihood ratio test of interaction, *P* = .75 at 34 weeks; eTable 4 in the Supplement), and the results were similar to those of the main study when we ran the regression models excluding medically indicated deliveries for perinatal death and SEN (eTables 4 and 5 in the Supplement). The results were again similar when we ran the logistic regression models excluding the twin pairs with 1 perinatal death and extreme birth weight discordance (eTable 6 in the Supplement).

## Discussion

The findings of this study suggest that birth at 37 completed weeks of gestation may be associated with optimal short- and long-term outcomes for twin infants. These data are in keeping with current clinical guidelines,^2,8^ which are informed by short-term outcomes but show for the first time, to our knowledge, that such a policy optimizes long-term outcomes as well. There is clear evidence that birth of twins before 37 weeks may be associated with an increased risk of perinatal mortality and the twin child having a record of SEN at school. In contrast to singletons, for whom the risk of SEN continues to decrease across all gestational ages until 41 weeks and then increases again at 42 weeks,^14^ among twins SEN is lowest at 37 weeks, with no statistically significant increase beyond that week. As stated previously, the sample size is limited in the later gestational weeks, and caution should be exercised in drawing firm conclusions from these findings.

By estimating how often twin pregnancies are complicated by 1 twin death occurring before the week of birth, these findings indicate that this situation is not a common problem and unlikely to alter the results of previous studies.

### Findings in the Context of Existing Literature

The results of the competing risk analysis were consistent with findings of other studies.^27^ However, those studies lacked information on adjustment for clustering in twins, and a proportion of the data were from randomized clinical trials and therefore different from population-based studies. A randomized clinical trial on the timing of twin birth is unlikely to be feasible given the large sample size required^6^; therefore, population-based studies remain the mainstay of investigation. Some studies have recommended birth of certain twin groups from 34 weeks; however, the present study does not provide any evidence of benefit from such a policy.^8,28^

To our knowledge, this is the first nationwide study to investigate long-term educational outcomes of twins across the range of gestational age categories. Overall, the population rate of SEN was higher in twins (13.77%) than has been previously estimated in singletons (4.90%),^14^ and this rate was higher in twins at each week of gestation (eTable 2 in the Supplement).

### Clinical and Research Implications

The findings of this study suggest that, in terms of short- and long-term outcomes, uncomplicated twin pregnancies should not be delivered before 37 completed weeks of gestation and that there is limited benefit of prolonging pregnancy thereafter. This information should be considered by women expecting twins and their caregivers when making decisions regarding timing of birth.

Several research priorities exist for twins, which require further investigation. As shown in the present study, preterm birth remains an important problem for twin pregnancies (39.2% of deliveries in this study were between 34 and 37 weeks of gestation), and at present there is no evidence of benefit of any interventions to reduce preterm birth in twins. Long-term school outcomes in twins according to birth order would be important to consider given the established increased risk of adverse outcomes for second twins.^29^

### Strengths and Limitations

This study has a number of strengths. Use of routinely collected population data ensured that every twin meeting the inclusion criteria was included, thus reducing selection bias. Both the obstetric and educational data were derived from routine population data, thus minimizing recall/ascertainment bias. The present study had several methodological analysis benefits over previous studies. First, this study accounted for the clustering in twins by using GEE analyses. Furthermore, by using adjusted sex- and gestation-specific birth weight centiles, the known interaction between birth weight and gestational age was adjusted for.^30,31^ Second, by carrying out a sensitivity analysis, the present study also investigated how often twin deliveries are complicated by 1 fetal death at an earlier gestational age than recorded.^32^ The inclusion of educational outcomes for twins is novel and provides long-term outcome data that are crucial to consider when planning the timing of birth.

The study also has limitations owing to the nature of routinely collected data. Missing covariates are a limitation and could reduce study power. Multiple imputation was used to counteract this problem, and the final results were compared with a complete case note analysis performed as a sensitivity analysis. Residual confounding is a potential limitation; 2 potential confounders that were unable to be addressed were chorionicity and conception status (conceived by assisted reproduction technologies). A sex-discordant analysis was performed to represent dichorionic pregnancies. Adjustment for those born using assisted reproduction technologies was not possible, but recent studies have suggested that there is no difference in perinatal outcomes between twins conceived naturally and those conceived with the use of assisted reproduction technologies.^33,34^ Medical indication for delivery is not recorded in SMR02; however, *ICD-10* codes for medical complications in pregnancy were identified to account for this. The sample size in some of the analyses is a limitation, especially in the group born after 38 weeks, which likely results from the policy of planned delivery around 37 to 38 weeks. The SEN analysis was limited by data linkage, and only sex-discordant twins could be used, thus reducing the sample size further; therefore, results of the SEN analysis may apply only to dichorionic twins.

## Conclusions

Twins born in Scotland between 1980 and 2015 were observed to have increased rates of perinatal mortality and SEN at school if delivered before 37 weeks of gestation. After 37 weeks, the risks of stillbirth and neonatal death may be balanced, and there may be no increased risk of SEN in deliveries beyond 37 weeks. Neonatal morbidity appeared to be lowest at 38 weeks of gestation; therefore, this study’s findings suggest that the optimal gestational age for birth of uncomplicated twin pregnancies is after 37 completed weeks.

## Notes

Supplement. **eFigure.** Prevalence of Special Educational Need by Gestation at Birth **eTable 1.** Perinatal Mortality at Each Gestation Category Compared to Remaining In Utero in Dichorionic Twins **eTable 2.** Differences in the Proportion of Special Educational Need (SEN) Between Twins and Singletons Overall and According to Gestation at Birth, Singleton Data Taken From MacKay et al **eTable 3.** Sensitivity Analysis: Complete Case Note Analysis (n = 23 762) **eTable 4.** Sensitivity Analysis of Perinatal Death in Nonmedically Indicated Deliveries (n = 38 225 for Nonmedically Indicated Deliveries) **eTable 5.** Sensitivity Analysis of SEN in Non-medicated Deliveries (n = 7,584 for Nonmedically Indicated Deliveries) **eTable 6.** Sensitivity Analysis: Removal of Cases Complicated by One Perinatal Death and Extreme Birth Weight Discordance (n = 382 Removed) **eReference**
