# Supplementary material for: Association of Gestational Age at Birth With Risk of Perinatal Mortality and Special Educational Need Among Twins
Source: JAMA Pediatr. 2020 Mar 9;174(5):1–9. doi: 10.1001/jamapediatrics.2019.6317 (PMC7063542; doi:10.1001/jamapediatrics.2019.6317)
Supplement: Supplement. — eFigure. Prevalence of Special Educational Need by Gestation at Birth eTable 1. Perinatal Mortality at Each Gestation Category Compared to Remaining In Utero in Dichorionic Twins eTable 2. Differences in the Proportion of Special Educational Need (SEN) Between Twins and Singletons Overall and According to Gestation at Birth, Singleton Data Taken From MacKay et al eTable 3. Sensitivity Analysis: Complete Case Note Analysis (n = 23 762) eTable 4. Sensitivity Analysis of Perinatal Death in Nonmedically Indicated Deliveries (n = 38 225 for Nonmedically Indicated Deliveries) eTable 5. Sensitivity Analysis of SEN in Non-medicated Deliveries (n = 7,584 for Nonmedically Indicated Deliveries) eTable 6. Sensitivity Analysis: Removal of Cases Complicated by One Perinatal Death and Extreme Birth Weight Discordance (n = 382 Removed) eReference [file jamapediatr-174-437-s001.pdf]

# Supplementary Online Content

Murray S, MacKay D, Stock S, Pell J, Norman J. Association of gestational age at birth with risk of perinatal mortality and special educational need among twins. *JAMA Pediatr.* Published online March 9, 2020. doi:10.1001/jamapediatrics.2019.6317

**eFigure.** Prevalence of Special Educational Need by Gestation at Birth

**eTable 1.** Perinatal Mortality at Each Gestation Category Compared to Remaining In Utero in Dichorionic Twins

**eTable 2.** Differences in the Proportion of Special Educational Need (SEN) Between Twins and Singletons Overall and According to Gestation at Birth, Singleton Data Taken From MacKay et al[1]

**eTable 3.** Sensitivity Analysis: Complete Case Note Analysis (n = 23,762)

**eTable 4.** Sensitivity Analysis of Perinatal Death in Nonmedically Indicated Deliveries (n = 38,225 for Nonmedically Indicated Deliveries)

**eTable 5.** Sensitivity Analysis of SEN in Nonmedicated Deliveries (n = 7,584 for Non-medically Indicated Deliveries)

**eTable 6.** Sensitivity Analysis: Removal of Cases Complicated by One Perinatal Death and Extreme Birth Weight Discordance (n = 382 Removed)

**eReference**

This supplementary material has been provided by the authors to give readers additional information about their work.

© 2020 Murray S et al. *JAMA Pediatrics.*

**eFigure.** Prevalence of Special Educational Need by Gestation at Birth

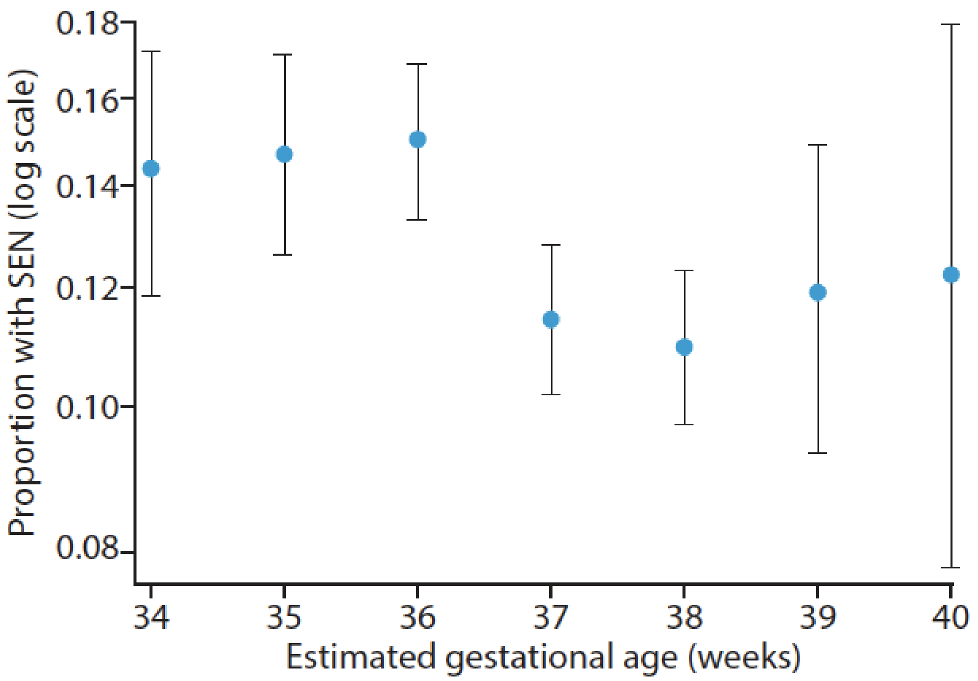


© 2020 Murray S et al. *JAMA Pediatrics.*

**eTable 1.** Perinatal Mortality at Each Gestation Category Compared to Remaining *In Utero* in Dichorionic Twins.

| | Ongoing pregnancies N[a] with outcome/total no in group (%) | Delivered N with outcome/total no in group (%) | OR[b] (95% CI[c]) | P value | Adjusted* OR (95% CI) | P value |
|---|---|---|---|---|---|---|
| 34-36 | 294/34698 (0.98) | 77/3527 (2.18) | 1.75 (1.26-2.44) | <0.001 | 1.87 (1.32-2.64) | <0.001 |
| 37-38 | 222/29952 (0.88) | 72/4746 (1.52) | 0.48 (0.29-0.80) | 0.005 | 0.59 (0.34-1.01) | 0.055 |

[a]N= number, [b]OR = odds ratio, [c]CI = 95% confidence intervals

*Adj. for maternal age & height, parity, baby sex, birth order, year of delivery, social class, birth weight centiles, smoking, height

© 2020 Murray S et al. *JAMA Pediatrics.*

**eTable 2.** Differences in the Proportion of Special Educational Need (SEN) Between Twins and Singletons Overall and According to Gestation at Birth, Singleton Data Taken From MacKay et al[1]

| Week of gestation | Proportion SEN twins N (%) (Non-medically indicated births) | Proportion SEN singletons N (%) (Non-medically indicated births) |
| --- | --- | --- |
| 33-36 | 395/2428 (16.27) | 1281/16754 (7.65) |
| 37 | 240/1847 (13.00) | 1217/18617 (6.54) |
| 38 | 210/1800 (11.67) | 2759/48810 (5.65) |
| 39 | 52/441 (11.79) | 3848/77217 (4.98) |
| >40 | 21/150 (14.00) | 10133/222943 (4.55) |
| Overall rate | 918/6666 (13.77) | 17784/362688 (4.90) |

© 2020 Murray S et al. *JAMA Pediatrics.*

**eTable 3.** Sensitivity Analysis: Complete Case Note Analysis (n = 23,762)

| Week of Gestation | Ongoing pregnancies N[a] with outcome/total no in group (%) | Delivered N with outcome/total no in group (%) | OR[b] (95% CI[c]) | P value | Adjusted* OR (95% CI) | P value |
|---|---|---|---|---|---|---|
| 34 | 387/39359 (0.98) | 85/3774 (2.25) | 2.32 (1.80-3.00) | <0.001 | 2.36 (1.56-3.58) | <0.001 |
| 35 | 302/34228 (0.88) | 85/5131 (1.66) | 1.89 (1.45–2.44) | <0.001 | 2.40 (1.65-3.50) | <0.001 |
| 36 | 199/26172 (0.76) | 103/8056 (1.28) | 1.69 (1.31-2.18) | <0.001 | 2.39 (1.58-3.59) | <0.001 |
| 37 | 122/15247 (0.80) | 77/10925 (0.70) | 0.88 (0.65-1.18) | 0.397 | 1.60 (0.93-2.75) | 0.087 |
| 38 | 49/5115 (0.96) | 73/10132 (0.72) | 0.75 (0.52-1.09) | 0.129 | 1.08 (0.43-2.73) | 0.868 |
| 39 | 20/1854 (1.08) | 29/3261 (0.89) | 0.82 (0.45-1.49) | 0.520 | 5.43 (0.15-190.1) | 0.315 |

[a]N= number, [b]OR = odds ratio, [c]CI = 95% confidence intervals *Adj. for maternal age & height, parity, baby sex, birth order, year of delivery, social class, birth weight centiles, smoking, height

© 2020 Murray S et al. *JAMA Pediatrics.*

**eTable 4.** Sensitivity Analysis of Perinatal Death in Non-medically Indicated Deliveries (n=38,225 for non-medically indicated deliveries)

| Week of gestation | All Twins<br>N in group, OR (95% CI), p value | Non-Medically indicated<br>N in group, OR (95% CI), p value | P value Interaction |
|---|---|---|---|
| 34 | 3774, 2.08 (1.42-3.06), <0.001 | 3527, 2.25 (1.66-3.05), <0.001 | 0.751 |
| 35 | 5131, 1.66 (1.12-2.47), 0.011 | 4746, 1.82 (1.34-2.48), <0.001 | 0.725 |
| 36 | 8056, 1.62 (1.08-2.41), 0.019 | 7123, 1.58 (1.17-2.13), 0.003 | 0.931 |
| 37 | 10925, 0.73 (0.44-1.21), 0.225 | 9516, 0.89 (0.63-1.26), 0.526 | 0.517 |
| 38 | 10132, 0.60 (0.31-1.17), 0.132 | 8,707, 0.76 (0.49-1.18), 0.228 | 0.558 |
| 39 | 3261, 0.88 (0.30-2.63), 0.822 | 2912, 0.74 (0.37-1.45), 0.381 | 0.784 |

© 2020 Murray S et al. *JAMA Pediatrics.*

**eTable 5.** Sensitivity Analysis of SEN in Nonmedicated Deliveries (n=7,584 for non-medically indicated deliveries)

| Week of gestation | All Twins<br>N in group, adj. OR (95% CI), p value | Non-Medically indicated<br>N in group, adj. OR (95% CI), p value |
|---|---|---|
| 34 | 780, 1.35 (1.14-1.82) | 616, 1.24 (0.91-1.71) |
| 35 | 1153, 1.35 (1.05-1.74) | 861, 1.32 (1.01-1.74) |
| 36 | 1795, 1.39 (1.11-1.74) | 1346, 1.31 (1.03-1.66) |
| 37 | 2564, REF | 2087, REF |
| 38 | 2423, 1.00 (0.80-1.24) | 2010, 0.94 (0.75-1.19) |
| 39 | 602, 1.02 (0.73-1.44) | 493, 0.89 (0.61-1.31) |
| >40 | 202, 1.16 (0.69-1.96) | 171, 1.14 (0.66-1.96) |

© 2020 Murray S et al. *JAMA Pediatrics.*

**eTable 6.** Sensitivity Analysis: Removal of Cases Complicated by One Perinatal Death and Extreme Birth Weight Discordance (n = 382 removed)

| Week of gestation | All Twins<br>Adjusted OR (95% CI) | BW discordant deaths removed<br>Adjusted OR (95% CI) |
|---|---|---|
| 34 | 2.59 (1.99-3.39) | 2.39 (1.63-3.49) |
| 35 | 2.12 (1.63-2.76) | 1.82 (1.24-2.68) |
| 36 | 1.99 (1.53-2.59) | 1.71 (1.24-2.35) |
| 37 | 1.10 (0.81-1.51) | 1.16 (0.76-1.75) |
| 38 | 0.92 (0.61-1.38) | 1.01 (0.60-1.71) |
| 39 | 0.77 (0.41-1.45) | 0.86 (0.36-2.07) |

© 2020 Murray S et al. *JAMA Pediatrics.*

**eReference**

1. MacKay DF, Smith GCS, Dobbie R, Pell JP. Gestational age at delivery and special educational need: retrospective cohort study of 407,503 schoolchildren. *PLoS Med*. 2010;7(6):e1000289. doi:10.1371/journal.pmed.1000289

© 2020 Murray S et al. *JAMA Pediatrics.*
